# The Effect of Grain Size on the Diffusion Efficiency and Microstructure of Sintered Nd-Fe-B Magnets by Tb Grain Boundary Diffusion

**DOI:** 10.3390/ma15144987

**Published:** 2022-07-18

**Authors:** Shuai Guo, Xiao Yang, Xiaodong Fan, Guangfei Ding, Shuai Cao, Bo Zheng, Renjie Chen, Aru Yan

**Affiliations:** 1 CISRI & NIMTE Joint Innovation Center for Rare Earth Permanent Magnets, Ningbo Institute of Material Technology and Engineering, Chinese Academy of Sciences, Ningbo 315201, China; dingguangfei@nimte.ac.cn (G.D.); caoshuai@nimte.ac.cn (S.C.); zhengbo@nimte.ac.cn (B.Z.); chenrj@nimte.ac.cn (R.C.); aruyan@nimte.ac.cn (A.Y.); 2 Key Laboratory of Magnetic Materials and Devices, Ningbo Institute of Material Technology and Engineering, Chinese Academy of Sciences, Ningbo 315201, China; yangxiaoworkhard@163.com; 3 University of Chinese Academy of Sciences, Beijing 100049, China

**Keywords:** grain size, diffusion efficiency, microstructure, sintered Nd-Fe-B magnets

## Abstract

The grain boundary diffusion process (GBDP) of heavy rare earth Tb is an effective method to improve the coercivity of Nd-Fe-B magnets, and the matrix grain size has a crucial effect on the diffusion efficiency and depth of the Tb element. In this work, magnets with different grain sizes have been fabricated using powder metallurgy to investigate the effect of grain size on Tb diffusion efficiency and the microstructure of Nd-Fe-B-type magnets. After the Tb diffusion process, the coercivity increment of the magnet with 4.9 μm large grain is 8.60 kOe, which is much higher than that of the magnet with 3.0 μm small grain (~5.90 kOe), which clearly demonstrates that the coercivity increment decreases as the grain size decreases. Microstructure analysis suggested that grain refinement significantly increases the total surface area, resulting in narrowing and discontinuity of the grain boundary phase (GBP). Therefore, as the channel for diffusion, the narrowing and discontinuity of the GBP are unfavorable for diffusion, resulting in a decrease in diffusion efficiency.

## 1. Introduction

Nd-Fe-B-based sintered magnets are widely used in many magnetic devices such as traction motors for hybrid or electronic vehicles and wind generators because of their excellent magnetic properties of high remanence and magnetic energy product [1,2,3]. However, the coercivity of the actual magnet is far below the intrinsic anisotropy field of the Nd_2_Fe_14_B phase (~7.3T), which has become the key factor limiting the application of Nd-Fe-B magnets [2,3,4]. In order to enhance the coercivity of sintered Nd-Fe-B magnets, the introduction of heavy rare earth (HRE, such as Dy and Tb) elements is an effective method due to the higher anisotropy fields of HRE_2_Fe_14_B phases. However, the resultant reduction in remanence is unavoidable because of the ferrimagnetic coupling between the HRE atoms and Fe atoms. At present, it is widely accepted that the coercivity of sintered Nd-Fe-B magnets is mainly detrimental to the reversal domain wall nucleation mechanism, and the defect in the surface of matrix grains is the main reason that the practical coercivity is lower than the intrinsic anisotropy field of the Nd_2_Fe_14_B phase [5,6,7,8]. Accordingly, introducing heavy rare earth (HRE) elements into the surface layer of the matrix phase by the grain boundary diffusion process (GBDP) has been proved as an effective method to enhance the coercivity of sintered Nd-Fe-B magnets with a slight sacrifice of remanence [9,10,11,12,13,14]. In the GBDP, the typical core–shell structure is formed, and the surface layer of the matrix phase exhibits a higher anisotropy field to suppress the nucleation of the reversal domain, thereby obtaining higher coercivity [15,16].

In addition, it is well known that the coercivity of sintered Nd-Fe-B magnets exhibits a large grain size dependence [17,18,19]. Grain refinement technologies such as He gas jet milling [20] and the HDDR (hydrogenation disproportionation desorption recombination) process [21] could effectively improve coercivity within a certain range. Furthermore, apart from directly affecting the coercivity of sintered Nd-Fe-B magnets, grain refinement also affects the distribution of the grain boundary phase (GBP). Since the Nd-rich GBP serves as the main channel for HRE elements’ diffusion during GBDP, the microstructure of the GBP has a large influence on the diffusion efficiency. Cao et al. [22] reported that higher RE content promoted Tb diffusion and further contributed to the higher coercivity when studying the influence of rare earth content on coercivity. In sintered Nd-Fe-B magnets, the distribution of the GBP is influenced not only by RE content, but also by the grain size. However, there has been scarce research so far on the relationship between grain size and diffusion efficiency. So, it is meaningful work to investigate the joint effect of grain size and the GBDP on coercivity. In this work, original magnets with different grain sizes were designed to systematically investigate the effect on the diffusion efficiency according to microstructure analysis.

## 2. Materials and Methods

### 2.1. Experimental Procedure

Commercial strip casting alloys with a nominal composition of (Pr_0.2_Nd_0.8_)_29_Cu_0.2_Al_0.05_Co_0.5_Ga_0.1_B_0.98_Fe_bal_ were used as the initial alloys. The strip casting alloys were subjected to a subsequent hydrogen decrepitation (HD) process and further jet milling (JM) in a nitrogen atmosphere. The different average particle sizes of 3.0 μm, 2.5 μm, 2.02 μm, and 1.76 μm were controlled by adjusting the parameter of the JM process. (Nd,Pr)H_x_ powders were prepared by hydrogenating the Nd-Pr alloy consisting of 80 wt.% Nd and 20 wt.% Pr under H_2_ pressure of ~ 200 kPa for 4 hrs at 400 °C. Subsequently, the prepared magnetic powders were mixed with 2 wt.% (Pr, Nd)H_x_ (Pr:Nd = 2:8, wt.%) powder. Then, the mixed powders were compacted and aligned under a magnetic field of 2.25 T, followed by cold isostatic compacting under a pressure of 150 MPa. The green compacts were each sintered at 1095 °C, 1080 °C, 1065 °C, and 1050 °C for 2 h in a vacuum followed by gas quenching. Then, the as-sintered magnets prepared by different powders were machined into cylinders with a diameter of 10 mm and a height of 4 mm for diffusion. The machined magnets were divided into two groups. The magnets in the test group were immersed in alcohol-based TbH_x_ suspension for 5 s and then dried in a N_2_ atmosphere. Then, the test group and control group were both treated with heat treatment at 900 °C for 2 h and annealed at 500 °C for 2 h.

### 2.2. Characterization and Analysis Methods

The average particle size of the JM powder was measured by a laser particle size analyzer, HELOS/RODOS-BR. The samples were cut into cylinders at a size of Φ10 mm × 4 mm, and the magnetic properties of the final annealed samples were measured by pulsed field magnetometry (Hirst PFM-14). The microstructures and elemental distribution of the magnets were observed by a scanning electron microscope (SEM) (Quanta FEG 250, FEI Company, Hillsboro, OR, USA) operating at 20 kV. The contents of Pr, Nd, Tb, and Fe in the core and shell of the matrix phase were obtained using energy-dispersive X-ray spectroscopy (EDS). The contents of Tb at different depths in the diffused magnets were detected using a glow discharge atomic emission spectrometer (GD-OES) (Spectruma Analytik GMBH 750HP, Hof, Germany).

## 3. Results and Discussion

### 3.1. Grain Size and Magnetic Properties

**Figure 1:**
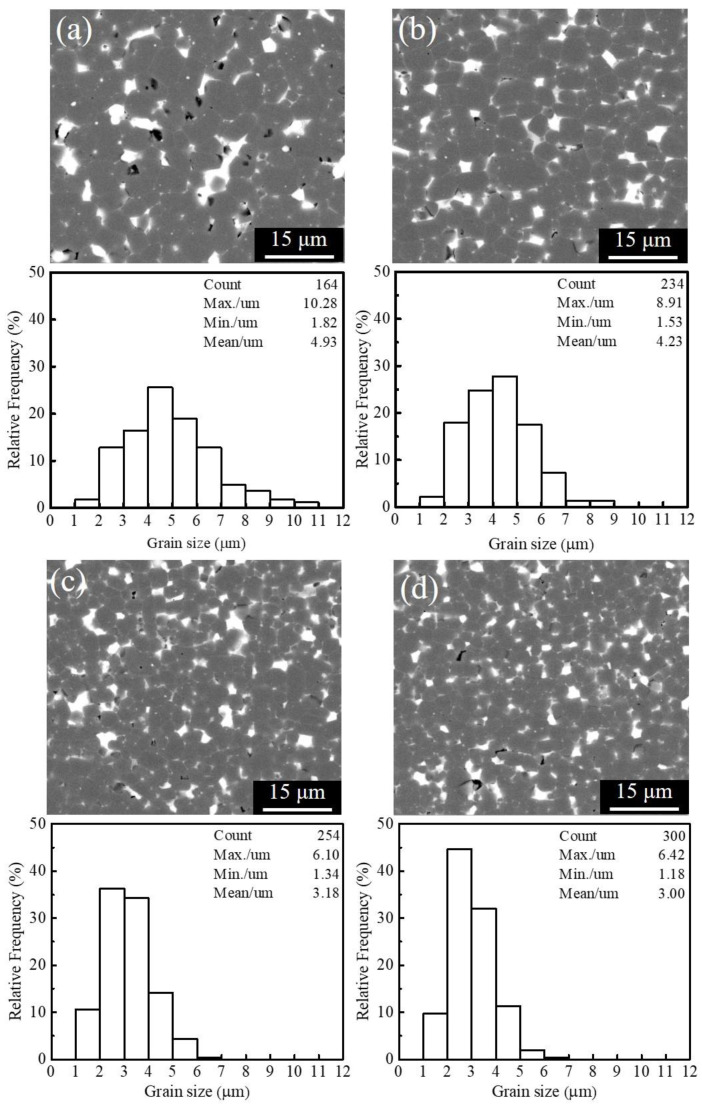
BSE SEM images and corresponding grain size distributions after sintering of magnetic powders with different particle sizes, (**a**) G4.9, (**b**) G4.2, (**c**) G3.2, (**d**) G3.0.

Figure 1 shows the backscatter electron (BSE) SEM images and corresponding grain size distributions after sintering of magnetic powders with different particle sizes. It is clear that after counting the dimensions of the matrix grains in every figure, the average grain sizes of the sintered magnets, fabricated by JM powders with different average particle sizes, are 4.93 μm, 4.23 μm, 3.18 μm, and 3.00 μm, which are marked as G4.9, G4.2, G3.2, and G3.0 magnets in the discussion below, respectively.

**Figure 2:**
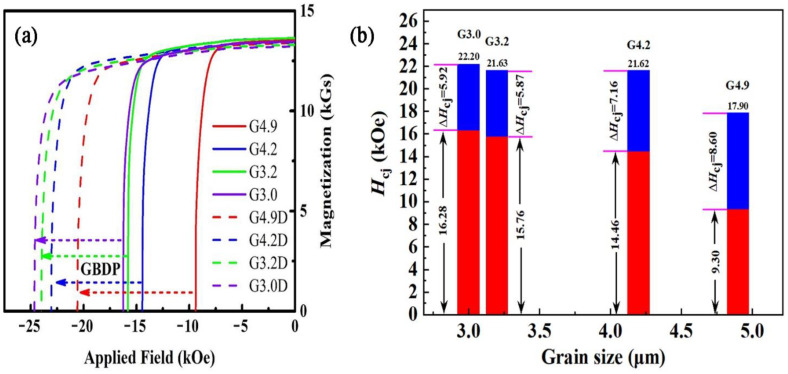
(**a**) The demagnetization curves of the original magnets (G4.9, G4.2, G3.2, and G3.0) and the diffused magnets (G4.9D, G4.2D, G3.2D, and G3.0D) with different grain sizes; (**b**) the coercivity of magnets with different grain sizes before and after the diffusion process (the red bars represent *H*_cj_ for the original magnets, and the blue bars represent the increments of *H*_cj_ after the diffusion process).

The demagnetization curves of original and diffused magnets with different grain sizes are shown in Figure 2, and the detailed values of coercivity, remanence, and maximum energy product derived from the demagnetization curves are listed in Table 1. The results show that the remanences and energy products of the magnets remain basically the same before and after the diffusion process, but the coercivities of the original magnets increase as the grain size decreases and have been enhanced significantly after diffusion. The more detailed coercivity change after diffusion is shown in Figure 2b, which demonstrates that the coercivity increment (Δ*H*_cj_ in Figure 2b) after diffusion decreases as the grain size decreases.

So, it could be concluded that the decrease in grain size is detrimental to diffusion efficiency. In addition, although the original coercivity of the G3.2 magnet is higher than that of the G4.2 magnet, the coercivities of these two magnets after diffusion are almost the same. Therefore, for commercial magnet fabrication, comprehensive consideration of grain refinement technology and the GBDP is necessary. Moreover, since the increase in coercivity of the magnet after the GBDP is derived from the Tb-rich shell structure, the difference in the coercivity increment should be related to the distribution of the shell structure.

### 3.2. Microstructure and Element Distribution

**Figure 3:**
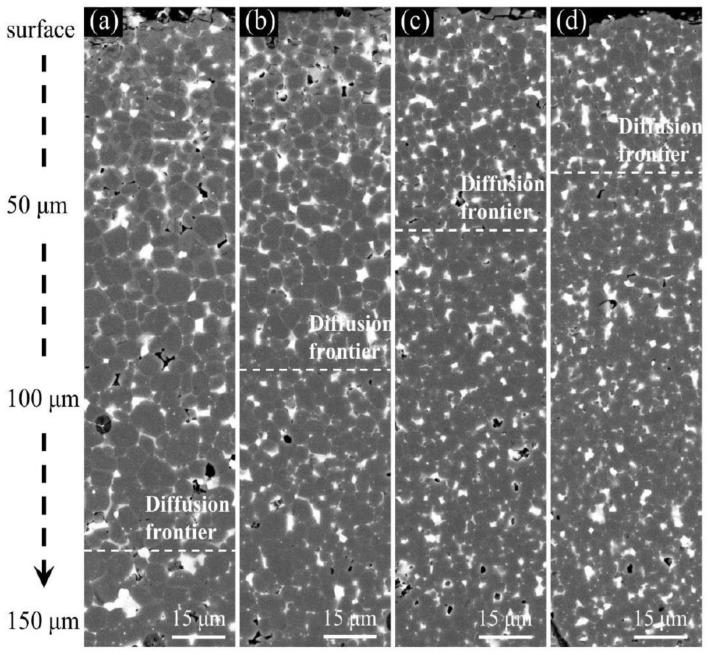
The longitudinal cross-sectional BSE-SEM images of (**a**) G4.9D, (**b**) G4.2D, (**c**) G3.2D, and (**d**) G3.0D diffused magnets.

Figure 3 shows the cross-sectional BSE SEM images of the magnets with different grain sizes after diffusion from the surface to about 150 μm depth. It is clear that a core–shell structure is formed in the matrix grains on the surface layer for the diffused magnets, and there are two phases with different contrast in the matrix grains after diffusing the Tb atoms. The brighter contrast in the shell area of the matrix grain corresponds to the Tb-rich (Pr,Nd,Tb)_2_Fe_14_B phase, while the darker contrast in the core area of the matrix grain corresponds to the Tb-lean (Pr,Nd)_2_Fe_14_B phase. In addition, the thickness of the Tb-rich shell decreases with the increase in diffusion depth for all four different grain size magnets. In the G4.9D magnet (Figure 3a), the Tb-rich shell structure, which is clearly visible in the BSE image surrounding the (Pr,Nd)_2_Fe_14_B core, is well performed. However, when the diffusion depth increases to about 150 μm, the Tb-rich core–shell structure becomes almost invisible. Actually, the core–shell structure still exists when the depth exceeds 150 μm, but the thickness of the Tb-rich shell is too small to see. The most interesting thing is that as the grain size decreases, the depth of the Tb diffusion area with visible core–shell structure decreases gradually. In the G4.2D magnet, the core–shell structure is obvious at a depth of about 100 μm, but in the G3.0D magnet, the apparent core–shell structure can only be found at a depth of less than 50 μm.

**Figure 4:**
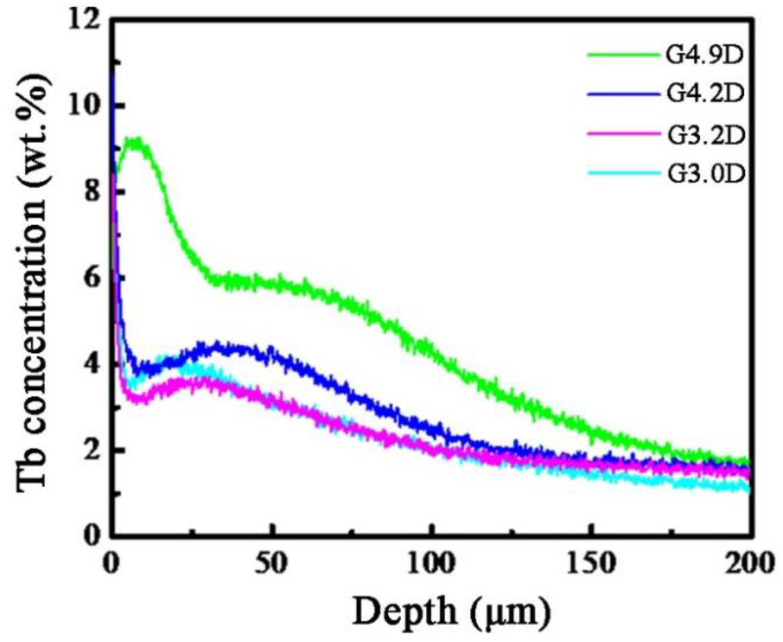
Tb contents as a function of depth from the surface of G4.9D, G4.2D, G3.2D, and G3.0D diffused magnets.

In order to further investigate accurate Tb content with increasing depth, the concentrations of Tb were obtained for G4.9D, G4.2D, G3.2D, and G3.0D diffused magnets, as shown in Figure 4. Affected by the diffusion dynamics, the distribution of Tb element in all magnets follows the law that the Tb concentration decreases gradually with the increase in diffusion depth. There exist different characteristics of Tb distribution in the magnets due to the different grain sizes. It is obvious that, under the same diffusion depth, Tb concentration in the surface area of the matrix grain for the large grain magnet is higher than that of the small grain magnet. However, as the diffusion depth increases, the concentration of Tb gradually becomes the same in all magnets. Particularly, the Tb concentration in the large grain magnet is still higher than that of the small grain magnet, while the diffusion depth exceeds 150 μm, which means that the large grain size facilitates the infiltration of Tb to the interior of the magnet.

**Figure 5:**
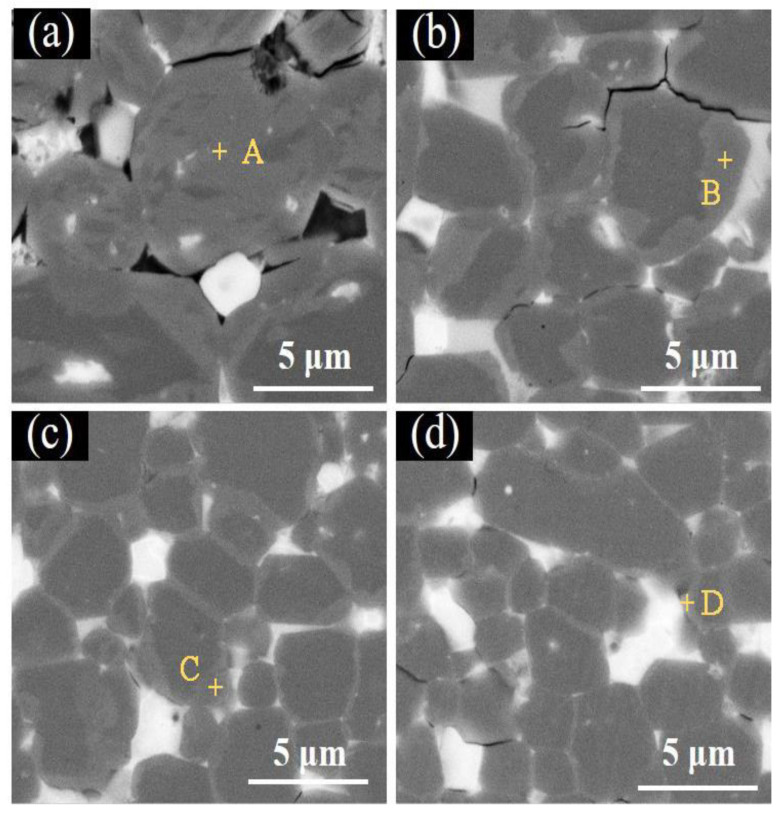
The BSE-SEM images at 15 μm from the surface of (**a**) G4.9D, (**b**) G4.2D, (**c**) G3.2D, and (**d**) G3.0D diffused magnets.

The BSE-SEM images at 15 μm from the surface of magnets are shown in Figure 5. This region is very close to the surface of the magnet, and the concentration of Tb in the grain boundary phase is relatively high. Therefore, the diffusion of Tb to the matrix phase is sufficient and the core–shell structure is obviously formed. However, the thickness of the shell structure in magnets with different grain sizes is not the same. It has been discovered that the thickness of the Tb-rich shell structure decreases with the decrease in grain size. In the G4.9D magnet, Tb has almost penetrated through the entire grain. However, in the G4.2D and G3.2D magnets, there exist distinct shell structures on the matrix grain’s surface and the Tb-rich shell in the G4.2D magnet is thicker than that in the G3.2D magnet. However, in the G3.0D magnet, Tb only concentrates in the superficial layer of the matrix phase and the thickness of the Tb-rich shell is much lower than that of the G4.2D and G3.2D magnets.

The contents of Tb in the shell region of the matrix grains in different magnets (points A–D in Figure 5) are shown in Table 2. It is clear that the Tb content in the shell region of the matrix grain gradually decreases with the increasing grain size, which is in accordance with the analysis of the core–shell structure in Figure 5. On the contrary, the contents of Pr and Nd in the shell region increase rapidly as the grain size decreases, which means that a small grain size prevents Tb diffusion to the interior of the magnets. In conclusion, the above results demonstrate that the diffusion depth of Tb atoms in Nd-Fe-B-type magnets has been restricted by the small grain size, and the detailed reason will be discussed below.

### 3.3. Diffusion Process Analysis

**Figure 6:**
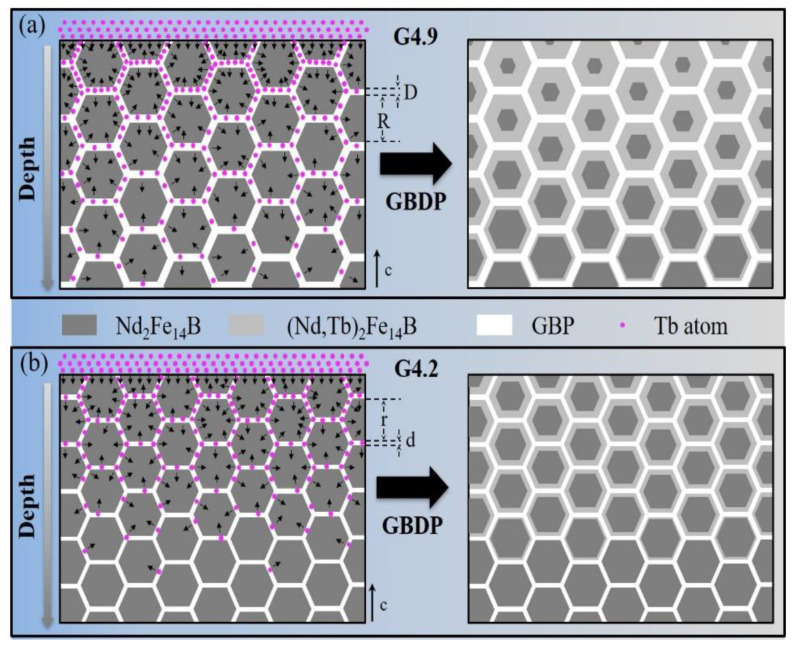
The schematic illustration of the diffusion process of the (**a**) G4.9 and (**b**) G4.2 magnets.

As is widely known, the GBP is the main channel for Tb diffusion, but the distribution of the GBP is closely related to the grain size. On the one hand, for spherical-like grains, the specific surface area of the single grain increases with the decrease in the grain volume; thus, the total surface area of the matrix grains increases as the grain size decreases. On the other hand, in sintered Nd-Fe-B-type magnets, the volume of GBPs for different magnets is basically the same. Therefore, the volume of GBP covering the unit surface region of the matrix grain surface reduces with the decrease in grain size, which leads to the GBP becoming thinner and more discontinuous. A schematic diagram for the grain boundary diffusion processes of magnets with different grain sizes is shown in Figure 6. The grain size of the G4.9 magnet (R) is larger than the grain size of the G4.2 magnet (r), so the GBP of the G4.9 magnet should be wider and more continuous than the GBP of the G4.2 magnet, that is to say, D > d. Because the GBP is the main channel for the Tb diffusion process, wider and more continuous GBP is beneficial to Tb diffusion [22,23]. In the G4.9 magnet, Tb is easier to diffuse into the interior of magnets along the GBP. With the grain size decreasing, the GBP becomes narrower and more discontinuous, and the grain boundary diffusion of Tb is insufficient; therefore, the diffusion depth is limited. At the same time, due to the Tb concentration in the GBP of the G4.9 magnet being higher than the G4.2 magnet, more Tb atoms diffuse into the main phase of the G4.9 magnet than into the G4.2 magnet. So, the shell thickness of the G4.9D magnet is larger than the G4.2D magnet, which is consistent with the SEM results.

## 4. Conclusions

In summary, the effect of grain size on Tb diffusion efficiency and microstructure was systematically investigated. After the diffusion process, the coercivity increment decreases as the grain size decreases, and the magnet with an average grain size of 4.9 μm has the largest coercivity increment of 8.60 kOe. Microstructure analysis suggests the variation in the coercivity increment is caused by the difference in grain size. When the grain size decreases, the specific surface area and total surface area of grains gradually increase, which leads to the GBP becoming narrow and discontinuous. Since the GBP is the main channel for Tb diffusion, the narrow and discontinuous GBP inhibits the Tb diffusion. Meanwhile, because of the higher Tb concentration in the GBP of magnets with larger grain sizes, the thickness of the shell structure decreases as the grain size decreases. Finally, the magnets with larger grain sizes have deeper diffusion depth and higher coercivity increments.

## Figures and Tables

**Table 1:** Coercivity, remanence, and maximum energy product of the magnets with different grain sizes before and after diffusion.

Sample	*H*_cj_ (kOe)	*B*_r_ (kG)	(*BH*)_max_ (MGOe)
G4.9	9.30	13.53	44.27
G4.2	14.46	13.64	45.49
G3.2	15.76	13.64	45.47
G3.0	16.28	13.45	44.30
G4.9D	17.90	13.58	45.35
G4.2D	21.62	13.51	44.83
G3.2D	21.63	13.38	44.04
G3.0D	22.20	13.28	43.36

**Table 2:** The elemental contents (wt.%) of the selected position in Figure 5 using EDS analysis.

Position	Pr	Nd	Tb	Fe
A	2.77	10.90	15.93	70.40
B	4.20	15.75	13.12	66.93
C	4.77	17.50	7.41	70.32
D	6.30	20.07	6.50	67.13

## Data Availability

The data presented in this study are available on request from the corresponding author.
